# Transition from Straight Lateral to Direct Anterior Approach in Hip Hemiarthroplasty: Preservation of Independent Living and Lower 1-Year Mortality

**DOI:** 10.3390/jcm15041533

**Published:** 2026-02-15

**Authors:** Jasper van Hees, Lambert C. E. Visser, Sharon Groen, Ellie B. M. Landman, Stijn A. A. N. Bolink

**Affiliations:** 1 Department of Orthopedics and Trauma Surgery, Deventer Hospital, 7416 SE Deventer, The Netherlands; s.groen.6@student.rug.nl (S.G.); e.landman2@dz.nl (E.B.M.L.); s.bolink@dz.nl (S.A.A.N.B.); 2 Department of Orthopedics, University Medical Center Groningen, 9713 GZ Groningen, The Netherlands

**Keywords:** femoral neck fractures, hemiarthroplasty, arthroplasty, replacement, hip, activities of daily living, postoperative complications, mortality

## Abstract

**Background/Objectives**: Hip hemiarthroplasty (HHA) for femoral neck fractures (FNFs) can be performed via the posterolateral approach (PLA), straight lateral approach (SLA) or direct anterior approach (DAA). However, the optimal approach remains unclear. This study evaluated mortality and return-to-home rates following an institutional transition from SLA to DAA. **Methods**: This retrospective observational cohort study included patients who underwent primary cemented unipolar hip hemiarthroplasty for FNF during a period of transition in surgical approach (2015–2023). Clinical outcomes between the straight lateral and direct anterior approach were compared. Primary outcomes were the mortality and return-to-home rates. Secondary outcomes included perioperative parameters and complications. A subgroup analysis was performed using Fracture Mobility Score (FMS) and Katz activities of daily living (ADL) index to compare functional outcomes. **Results**: Over a 9-year period, a total of 762 HHA were performed, of which 411 SLA and 333 DAA. Mortality at 90 days (14.1% vs. 8.7%, *p* = 0.029) and 1 year (26.5% vs. 17.7%, *p* = 0.005) were significantly higher in the SLA group. Among patients living at home preoperatively, return-to-home after surgery was lower for SLA compared to DAA (23.2% vs. 41.4%, *p* < 0.001). In terms of complications, SLA had significantly lower rates of periprosthetic joint infections (SLA *n* = 6 (1.5%) vs. DAA *n* = 15 (4.6%), *p* = 0.024). The decline in Katz ADL score at three months was significantly greater in the SLA group than in the DAA group (ΔKatz ADL −0.73 ± 1.57 vs. −0.11 ± 1.60, *p* = 0.036). **Conclusions**: Transitioning from SLA to DAA in HHA was associated with improved preservation of independent living, higher return-to-home rates and lower 90-day and 1-year mortality. However, DAA was also associated with higher rates of PJI.

## 1. Introduction

Hip hemiarthroplasty (HHA) is widely used in the surgical management of femoral neck fractures (FNFs) [1]. FNFs are frequent among elderly, primarily due to falls, and are associated with substantial morbidity and mortality, with a reported 1-year mortality of 22–33% following surgery [2,3]. When left untreated, 30-day mortality of 31–36% is reported [4,5]. This places a heavy burden on healthcare systems worldwide [6,7].

Multiple surgical approaches can be used to perform HHA, including the posterolateral approach (PLA), straight lateral approach (SLA), and direct anterior approach (DAA). Currently, the SLA and PLA are the most used worldwide [8].

However, over the past two decades the DAA has gained popularity [9,10]. This rising popularity of the DAA for HHA has also been observed in the Netherlands. Although the PLA and SLA still account for the majority of HHA procedures, together representing over 75% of cases, a clear national shift in surgical approach has emerged. Data from the Dutch Arthroplasty Register (LROI) show that the use of the DAA in HHA increased from 6.6% in 2018 to 15.5% in 2024. This growth occurred mainly at the expense of the PLA. PLA use declined steadily from 51% in 2014 to 40.7% in 2023. The use of approaches besides DAA and PLA remained relatively stable over this period [11].

The rise in popularity of the DAA could be attributed to the growing body of evidence highlighting its potential clinical advantages. DAA is associated with less postoperative pain, reduced operation time, shorter length of stay, and smaller haemoglobin drop after surgery when compared to SLA. Furthermore, a trend towards fewer complications when a transition was made from SLA to the DAA is seen [12,13,14,15,16,17]. As a result, elderly patients are expected to have higher discharge-to-home rates and reduced reliance on walking aids during the early postoperative period [12,16,18]. The expected functional improvements have already been observed in some studies that demonstrated earlier postoperative mobilisation and superior activities of daily living (ADL) at one day postoperatively following DAA [12,19,20,21,22]. Such potential benefits could be especially important in patients suffering from a FNF, who are often frail and burdened by multiple comorbidities.

Evidence regarding differences in mortality between surgical approaches in HHAs remains limited. Several factors likely contribute to the limited exploration of this topic to date. First of all, DAA has historically been more widely adopted in elective arthroplasty than in emergency fracture surgery [11,13]. Its use in HHA has only increased over the last decade. As a result, comparative mortality data in patients with femoral neck fractures remains scarce [20]. Furthermore, assessing mortality differences is methodologically challenging, as survival in this population is heavily influenced by factors such as frailty and comorbidities [2,3]. These factors may obscure the independent effect of surgical technique. Nevertheless, the literature comparing approaches in total hip arthroplasty (THA) suggests that the DAA may be associated with reduced 1-year mortality and improved functional outcomes compared with PLA [23,24,25]. However, this association has not yet been observed when compared to the SLA [15,26].

Based on the potential benefits, our level 2 trauma centre decided to transition from SLA to DAA in HHA for FNFs. This study primarily aims to evaluate the mortality and return-to-home rates resulting from this transition. Secondary, differences in clinical and functional outcomes are evaluated.

## 2. Materials and Methods

This retrospective observational cohort study within a level 2 trauma centre was approved by the institutional review board (nr. ME-14). All patients who underwent HHA within the transition period were screened for eligibility using an institutional hip fracture database. The transition period covered the period from 1 January 2015 to 31 December 2023. During this period the use of the straight lateral approach steadily decreased, while the use of the direct anterior approach increased, resulting in a near-complete shift to the direct anterior approach. All HHAs during this time period were performed by eight experienced board-certified trauma surgeons with at least 10 years of experience in hip fracture management. Patients were included if they underwent primary cemented unipolar HHA (Original Muller Stem, Zimmer Biomet) for an intracapsular femoral neck fracture. Patients were excluded if they had a history of any type of ipsilateral hip surgery or if an approach other than SLA or DAA was performed.

### 2.1. Straight Lateral Approach

The SLA was conducted with the patient positioned in a lateral decubitus position. A skin incision was made directly over the greater trochanter, after which the tensor fascia lata (TFL) was opened longitudinally. The aponeurotic attachments of the gluteus medius and minimus muscles were released at the anterior third of their trochanteric insertion point. A T-shaped incision was then made in the anterior capsule to access the hip joint. Following placement of the prosthetic components and hip reduction, both the anterior capsule and the gluteal muscle attachments were repaired using sutures.

### 2.2. Direct Anterior Approach

The DAA was performed with the patient in a supine position. An oblique incision was created along the trajectory of the TFL muscle. Following the initial incision, the TFL fascial layer was opened in a longitudinal direction, with subsequent blunt dissection carried out in the interval between the TFL and sartorius muscle. Lateral circumflex femoral artery branches were located and cauterised. The anterior hip capsule was then visualised and partially excised. Following femoral neck osteotomy and head extraction, the femoral and superior capsular portions were released, with the additional release of the conjoined tendon at the greater trochanteric saddle to enable appropriate elevation and exposure of the proximal femoral shaft. For optimal visualisation of the femoral canal, the operative limb was positioned in adduction and external rotation beneath the opposite leg in a figure-4 configuration, with bilateral hip hyperextension achieved through table flexion.

### 2.3. Outcomes

Baseline characteristics of included patients were extracted from the electronic patient files using a chart review. Characteristics included age, sex, pre-injury residential location (home or long-term care facility), body mass index (BMI, in kg/m^2^), and American Society of Anaesthesiologists (ASA) physical status classification, as well as any history of co-morbidities at admission, such as chronic obstructive pulmonary disease (COPD), dementia (including Alzheimer’s disease, vascular dementia, or previous reported cognitive deterioration), and osteoporosis.

The primary outcomes of this study were mortality and the return-to-home (RtH) rates. Mortality was determined during admission, at 30 days, 90 days and 1 year post-surgery. RtH was defined as returning to a residential location with no characteristic of long-term care facilities at the moment of discharge. Perioperative and postoperative care pathways with a centralised transfer department for patient flow and inter-institutional coordination remained consistent throughout the study period. Post-discharge timing of RtH was not systematically recorded, as such follow-up fell outside the scope of routine hospital care documentation using the national standardised Dutch Hip Fracture Audit Questionnaire.

Secondary outcomes included duration of surgery (DoS), length of stay after surgery (LoS), duration of admission (days), blood loss (mL), haemoglobin (Hb) drop, and HHA-related complications. Complications were systematically categorised as periprosthetic joint infections (PJI), revision surgery, periprosthetic fractures, prosthetic hip joint dislocations, and conversion arthroplasty.

PJI were diagnosed according to the 2021 European Bone and Joint Infection Society (EBJIS) criteria [27]. Revision surgery was defined as any surgical procedure requiring reopening of the original surgical incision with either retention or exchange of the hemiarthroplasty components. This included debridement, antibiotics, and implant retention (DAIR) procedures as well as periprosthetic fracture fixation. Conversion arthroplasty was defined as replacement of the HHA with a THA and was considered a distinct category separate from revision surgery.

The subgroup analysis represented the subset of patients for whom complete functional data were available. Predefined subgroup analyses were conducted in patients with Fracture Mobility Score (FMS) and Katz activities of daily living (Katz ADL) data to evaluate functional outcomes according to surgical approach.

The FMS was a validated 5-point scale evaluating mobility (1 = fully independent, 5 = completely immobile), used to assess pre-injury and 3-month postoperative functional status [28]. FMS at admission was scored by an emergency department nurse or geriatrician, while 3-month follow-up scores were assessed by the orthopaedic trauma surgeon or advanced practice nurse.

The Katz ADL evaluated independence in six basic self-care tasks (bathing, dressing, toileting, transferring, continence, feeding), scored as 0 (dependent) or 1 (independent) [29,30]. Katz ADL was used to assess preoperative functional status and 3-month postoperative functional status. Preoperative scores were obtained from admission records and the first geriatric assessment, while postoperative scores were obtained during follow-up by the orthopaedic trauma surgeon or advanced practice nurse.

### 2.4. Statistical Analysis

Categorical variables were presented as absolute numbers and percentages. Continuous variables were presented as mean and standard deviation. Survival analysis will be performed using Kaplan–Meier curves for visual inspection and group differences in survival probability will be assessed using log-rank test. For normally distributed data, group comparisons and subgroup comparisons were performed using independent samples *t*-test. For non-normally distributed data, the Mann–Whitney *U* test was used. Categorical variables were compared using the Chi-square test or Fisher’s exact test, as appropriate. For all tests, significance was set at a two-tailed *p* < 0.05. Statistics were performed in IBM SPSS Statistics, Version 29.0 (IBM Corp., Armonk, NY, USA, Released 2022).

### 2.5. Post Hoc Sensitivitiy Analysis

To evaluate the robustness of our findings and assess potential temporal trends related to learning curve effects or gradual change in perioperative management, a post hoc sensitivity analysis was performed. Outcomes were compared between the first 100 consecutive DAA cases and the last 100 consecutive DAA cases during the study period. In-hospital mortality, 30-day mortality, 90-day mortality, 1-year mortality, return-to-home discharge rates, and periprosthetic joint infection (PJI) rates were compared using Fisher’s exact test. To control for type I error inflation from multiple comparisons, a Holm–Bonferroni sequential correction was applied.

## 3. Results

**Figure 1:**
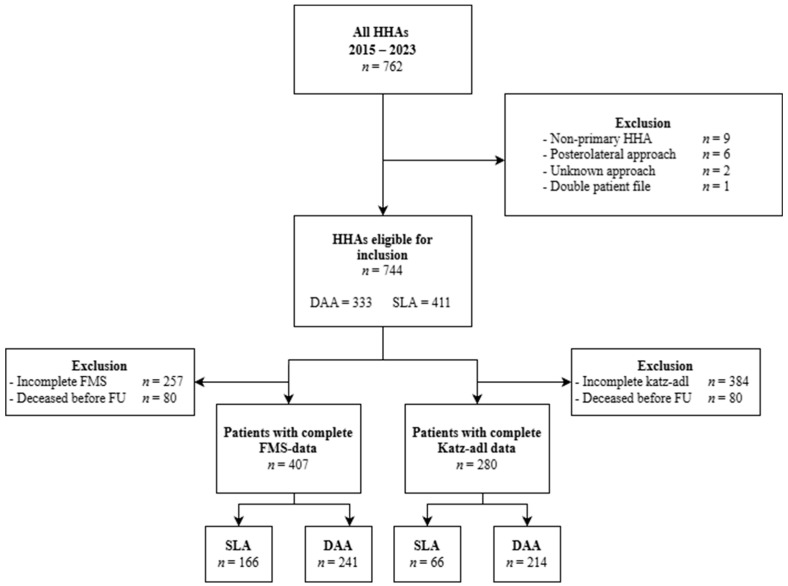
Flowchart of patient inclusion with subgroup selection.

**Figure 2:**
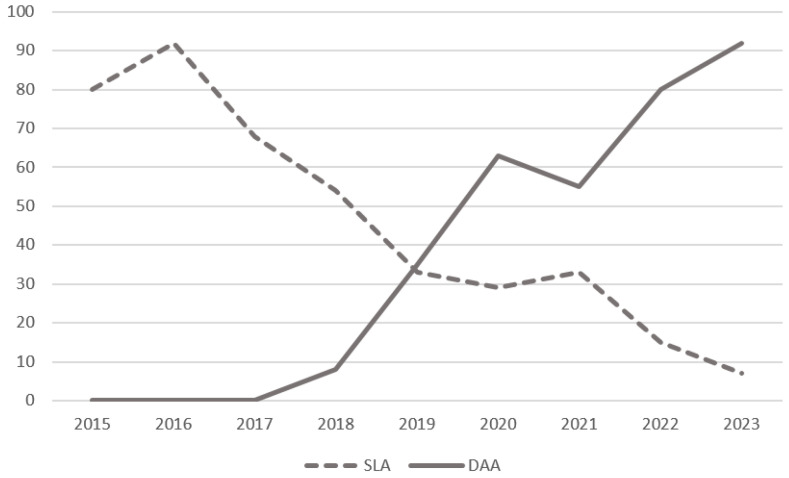
The course of the number of surgical approaches performed per year.

A total of 744 patients were included in this study, 333 treated via the direct anterior approach (DAA) and 411 via the standard lateral approach (SLA) (Figure 1). Over time, the use of the SLA gradually declined while the use of the DAA increased correspondingly, resulting in a clear crossover trend when plotted over time (Figure 2).

Baseline characteristics differed between groups on dementia (SLA; *n* = 96 (23.4%) vs. DAA; *n* = 55 (16.6%), *p* = 0.028) and living at home prior to admission (SLA; *n* = 267 (65%) vs. DAA; *n* = 257 (77.2%), *p* < 0.001). Age, gender, BMI, ASA-grade, and incidence of the co-morbidities like COPD, diabetes type I or II, and osteoporosis were comparable between groups (Table 1).

### 3.1. Primary Outcomes

**Figure 3:**
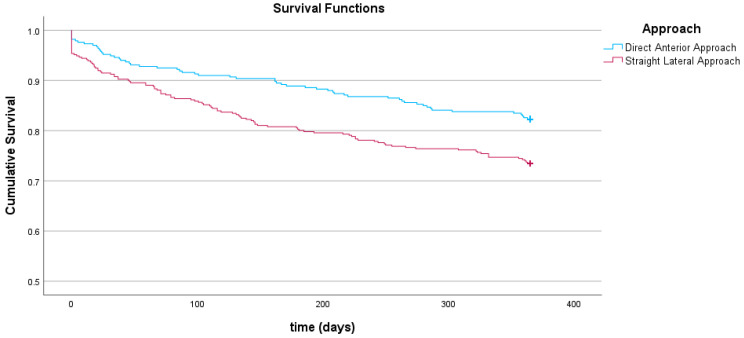
Kaplan–Meier curve illustrating one-year survival between different surgical approach.

Visual inspection of the Kaplan–Meier curve suggests a higher probability of survival up to 1 -year for the DAA (Figure 3). A log-rank test compared survival distribution between SLA and DAA and revealed a significant difference in survival in favour of the DAA after 1 year (X^2^ = 8.551, *p* = 0.003). No significant differences were observed between SLA and DAA during admission (SLA *n* = 18 (4.4%) vs. DAA *n* = 6 (1.8%), *p* = 0.060) or at 30 days postoperatively (SLA *n* = 37 (9.0%) vs. DAA *n* = 19 (5.7%), *p* = 0.095.). However, 90-day postoperatively mortality (SLA *n* = 58 (14.1%) vs. DAA *n* = 29 (8.7%), *p* = 0.029) and 1-year mortality were significantly higher in the SLA group compared to the DAA group (SLA *n* = 109 (26.5%) vs. DAA *n* = 59 (17.7%), *p* = 0.005) (Table 2).

**Figure 4:**
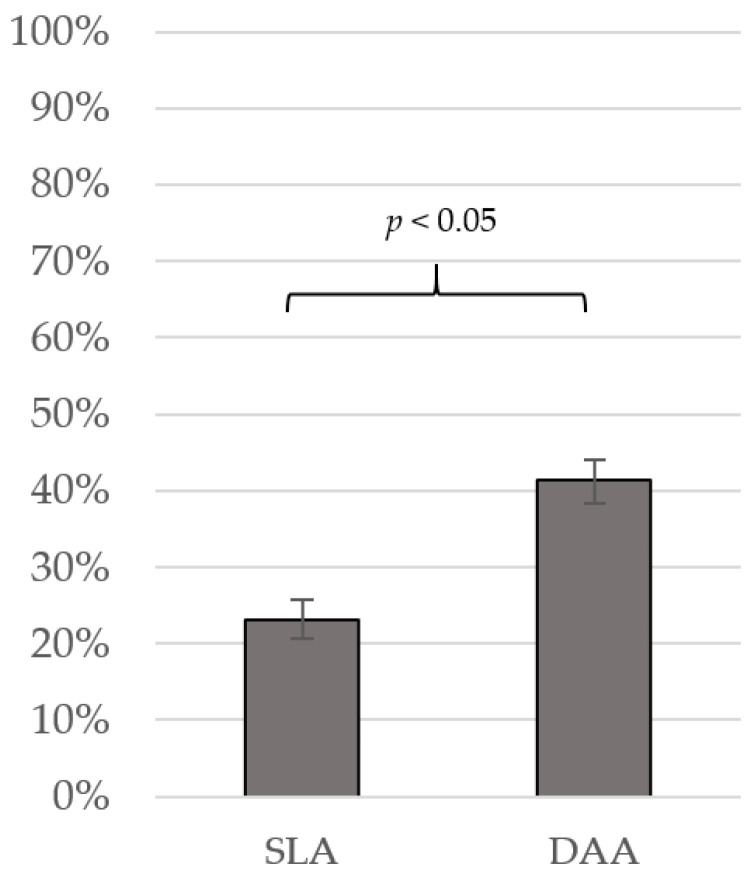
Return-to-home rates for straight lateral approach (SLA) and direct anterior approach (DAA). Error bars represent standard error.

For patients who lived at home preoperatively and did not decease during admission, the return-to-home rate at discharge was significantly lower for SLA when compared with DAA (SLA 60 (23.2%) vs. DAA 104 (41.4%, *p* < 0.001)) (Table 2) (Figure 4).

### 3.2. Secondary Outcomes

When comparing the duration of surgery between groups, no significant difference was observed (SLA 68.6 ± 16.9 min vs. DAA 69.5 ± 14.5 min, *p* = 0.445). The length of stay after surgery was comparable between SLA and DAA (5.80 ± 3.46 days vs. 5.58 ± 4.09 days, *p* = 0.440). Similarly, the total duration of stay demonstrated no significant difference (7.65 ± 3.86 days vs. 7.53 ± 4.35 days, *p* = 0.688). Blood loss (SLA 285.9 ± 131.6 mL vs. DAA 296.0 ± 138.8 mL, *p* = 0.344) and haemoglobin drops were comparable (SLA −0.93 ± 0.70 mmol/L vs. DAA −0.91 ± 0.69 mmol/L, *p* = 0.651) (Table 3).

When comparing HHA-related complications, it appeared that the SLA group had significantly lower rates of periprosthetic joint infections (SLA *n* = 6 (1.5%) vs. DAA *n* = 15 (4.6%), *p* = 0.024). The rates of revision surgery (SLA *n* = 9 (2.3%) vs. DAA *n* = 16 (4.9%), *p* = 0.067); periprosthetic fractures (SLA *n* = 2 (0.5%) vs. DAA *n* = 2 (0.6%), *p* = 1.00); hip joint dislocation (SLA *n* = 6 (1.5%) vs. DAA *n* = 3 (0.9%), *p* = 0.521); and conversion surgery to total hip arthroplasty (THA) were comparable (SLA *n* = 1 (0.3%) vs. DAA *n* = 2 (0.6%), *p* = 0.594) (Table 3).

### 3.3. Subgroup Analysis

For the subgroup analysis, 407 HHAs with complete mobility scores (SLA *n* = 166; DAA *n* = 241) and 280 HHAs with complete Katz ADL scores (SLA *n* = 66; DAA *n* = 214) were included. Demographic characteristics for the subgroup of patients with complete preoperative and follow-up for the FMS and Katz scores were comparable for all baseline characteristics (Table 4).

**Figure 5:**
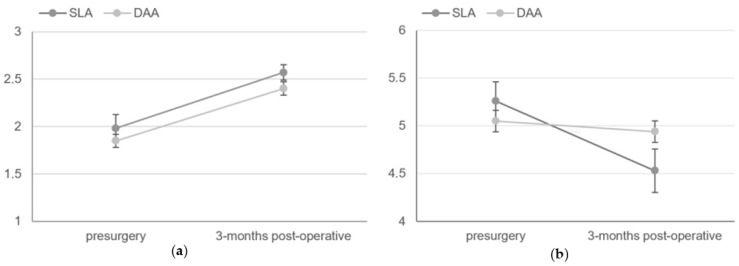
Pre-surgery and follow-up scores of (**a**) functional mobility scores (FMS) and (**b**) Katz activities of daily living (Katz ADL). Error bars represent standard error.

Preoperative FMS did not significantly differ between groups (SLA 1.98 ± 1.18 vs. DAA 1.85 ± 1.07, *p* = 0.243). At 3 months postoperatively, FMSs were comparable between groups (SLA 2.57 ± 1.03 vs. DAA 2.40 ± 1.11, *p* = 0.106) with a similar decline in FMSs (ΔFMS) between approaches (ΔSLA = 0.60 ± 1.06 vs. ΔDAA 0.54 ± 1.10, *p* = 0.822) (Figure 5) (Table 5).

Preoperative Katz ADL did not significantly differ between groups (SLA 5.26 ± 1.63 vs. DAA 5.05 ± 1.65, *p* = 0.250). At 3 months postoperatively, Katz ADL scores decreased in both groups with no significant differences between approaches (SLA 4.53 ± 1.84 vs. DAA to 4.94 ± 1.66, *p* = 0.063). The magnitude of decline in Katz ADL (ΔKatz ADL) differed significantly between groups, with a greater decrease observed in SLA compared with DAA (SLA −0.73 ± 1.57 vs. DAA −0.11 ± 1.60, *p* = 0.036) (Figure 5) (Table 5).

### 3.4. Post Hoc Sensitivity Analysis

In the post hoc sensitivity analysis, no statistically significant differences were observed. Periprosthetic joint infection rates were comparable between the early and late cohorts (4.0% vs. 5.0%, *p* = 1.00). Mortality rates did not differ significantly at any time point: in-hospital mortality (1.0% vs. 3.0%, *p* = 0.621), 30-day mortality (8.0% vs. 3%, *p* = 0.213), 90-day mortality (11.0% vs. 5.0%, *p* = 0.191), and 1-year mortality (18.0% vs. 11.0%, *p* = 0.228). Return-to-home discharge rates were comparable between groups (37.5% vs. 42.0%, *p* = 0.622).

## 4. Discussion

This study evaluated the potential benefits of DAA in HHA compared to SLA and found a significantly smaller decline in ADL dependency at 3 months postoperatively, when DAA was performed. Secondly, in patients who lived independently at home prior to surgery, DAA was associated with a higher return-to-home rate. Furthermore, this study demonstrated lower mortality at both 90 days and 1 year for DAA.

Although differences in mortality during hospital admission and at 30 days postoperatively were not significant, at 90-day and 1-year postoperative mortality rates were significantly lower for the DAA. These findings were in contrast with the existing literature where no clear difference in mortality between SLA and DAA has been observed in HHA [13,14]. The decreased 90-day and 1-year mortality observed for DAA may be partially explained by baseline differences between groups, as a higher proportion of DAA patients were living independently before surgery and there were fewer cases of dementia. These factors are known predictors of postoperative mortality and functional outcome after hip fracture surgery [31,32,33]. The association between DAA and improved survival observed in this study is noteworthy; however, causality cannot be established.

We observed a significantly higher rate of patients returning to home without the need for rehabilitation or long-term care facilities following the transition to DAA. Furthermore, a smaller decline in ADL independence at three months postoperatively was observed following DAA. These findings suggested that the DAA resulted in less functional decline during the early postoperative period and underlines its muscle-sparing character, which accords with the existing literature [12,15]. Moreover, a recent study in THA indicated that the anterior approach may help protect against nursing facility discharge and contributes to a decreased length of stay [34].

In the subgroup analysis, mobility and activities of daily living were evaluated using international validated questionnaires. Preoperative and postoperative mobility scores were comparable between DAA and SLA. The pre- to postoperative difference in Katz ADL demonstrated a significantly larger decline for SLA. This might indicate a functional advantage for the anterior approach, possibly due to reduced soft tissue damage resulting in faster early mobilisation [15,26].

The secondary outcomes of the current study aimed to further evaluate complications after transitioning from SLA to DAA. The length of stay, duration of surgery, blood loss, and postoperative haemoglobin drop were comparable between approaches, consistent with the existing literature. These factors were therefore unlikely to explain the observed differences in the primary outcomes.

The incidence of PJI was higher in the DAA group. This aligned with previous reports indicating that the DAA may be technically more demanding and associated with a steeper learning curve, potentially increasing the risk of wound-related complications [35,36]. The post hoc sensitivity analysis revealed no significant differences in PJI rates, suggesting that the higher incidence of PJI in the DAA group was unlikely to be attributed to the surgical learning curve alone. There were no demographic explanations for the PJI incidence, as known risk factors were comparable between groups (age, ASA, DM type II, BMI) and were even more favourable in the DAA group compared to the SLA group (e.g., dementia) [3,37].

This study has multiple notable strengths. First, the single-centre design ensured uniformity in perioperative protocols, anaesthetic management, and rehabilitation pathways, minimising institutional variation that often confounds multi-centre studies. Second, the gradual transition from SLA to DAA created a well-defined natural experiment, allowing direct comparison of both approaches within the same institutional context and surgical team during a clearly delineated period. Third, the cohort size of this single-centre study was relatively large in comparison with prior studies, providing higher statistical power to detect clinically relevant differences in outcomes [16,17,19,26]. Lastly, validated outcome measurement tools such as the fracture mobility score (FMS) and Katz ADL index were used, offering a multidimensional and clinically relevant evaluation of postoperative function.

Several limitations of the present study must be acknowledged. Most importantly, the lack of adjustment for baseline confounders represented the primary methodological limitation of this study. Patients in the DAA and SLA groups differed significantly in prognostic factors, particularly the prevalence of dementia and pre-fracture living status. In the absence of adjustment for these differences, the internal validity of the results may be affected. Additionally, our dataset did not capture whether patients had previously undergone THA of the contralateral hip, which may influence both fracture risk and postoperative outcomes. Given the growing prevalence of THA in the elderly population, the absence of these data represented a potential confounder [38]. As a retrospective study, the analysis is subjected to inherent limitations, including the potential for unmeasured bias and confounding. Reliance on chart reviews of electronic patient files may have introduced misclassification. Moreover, a substantial proportion of patients lacked complete FMS or Katz ADL data, which can be unfortunately common in this vulnerable elderly population, limiting the robustness of functional outcome analyses. Lastly, the single-centre setting, while ensuring internal consistency, may limit generalisability to other institutions with different surgical expertise or patient populations.

The assessment battery remained relatively limited to two functional domains: mobility (FMS) and activities of independent living (Katz ADL). Future research should incorporate more comprehensive patient reported outcome measures (PROMs), performance-based physical assessments, activity monitoring, and more complete follow-up data collection. Larger prospective randomised controlled trials and multi-centre collaborations are needed to confirm these findings, clarify causal relationships between surgical approaches and outcomes, account for learning curve effects, and improve the generalisability of results across diverse healthcare settings and patient populations.

## 5. Conclusions

In conclusion, this retrospective study shows that transitioning from SLA to DAA in HHA may be associated with both advantages and disadvantages. Patients treated with DAA appeared to better preserve their pre-fracture functional status, facilitating an earlier return to home. In addition, lower mortality rates at 90 days and 1 year were observed in DAA. However, the use of DAA during the transition period was also associated with a higher incidence of periprosthetic joint infections compared with SLA.

## Figures and Tables

**Table 1:** Demographic data.

Characteristics	SLA	DAA	*p*-Value
Number of HHAs	411	333	-
Age ^a^ (years)	82.2 ± 7.62	81.9 ± 7.40	0.669 ^c^
Gender, Female ^b^	283 (68.9%)	217 (65.2%)	0.308 ^d^
BMI ^a^ (kg/m^2^)	24.7 ± 4.02	24.7 ± 3.53	0.993 ^c^
ASA grade ^b^			0.295 ^e^
• ASA I	8 (2.0%)	5 (1.5%)	
• ASA II	180 (44.6%)	135 (40.7%)	
• ASA III	198 (49.0%)	183 (55.1%)	
• ASA IV	18 (4.5%)	9 (2.7%)	
Co-morbidities			
• COPD ^b^	43 (10.5%)	40 (12.0%)	0.559 ^d^
• Dementia ^b^	96 (23.4%)	55 (16.6%)	0.028 ^d,f^
• DM I/II ^b^	88 (22.7%)	58 (17.4%)	0.082 ^d^
• Osteoporosis ^b^	116 (28.2%)	94 (28.2%)	1.00 ^d^
Living at home prior to admission	267 (65.0%)	257 (77.2%)	<0.001 ^d,f^

SLA, straight lateral approach; DAA, direct anterior approach; HHAs, hip hemiarthroplasties; BMI, body mass index; ASA, American Society of Anesthesiologist;COPD, Chronic obstructive pulmonary disease; DM I/II, diabetes mellitus—type I and II. ^a^ Data are given as mean and standard deviation. ^b^ Data expressed as frequency *n* (%). ^c^ Independent *t*-test. ^d^ Fisher’s exact test. ^e^ Chi-square test. ^f^ Statistically significant difference.

**Table 2:** Mortality and return-to-home rates.

	SLA (*n* = 411)	DAA (*n* = 333)	*p*-Value
Mortality ^a^			
• During Admission	18 (4.4%)	6 (1.8%)	0.060 ^c^
• 30-day	37 (9.0%)	19 (5.7%)	0.095 ^c^
• 90-day	58 (14.1%)	29 (8.7%)	0.029 ^c,d^
• 1-year	109 (26.5%)	59 (17.7%)	0.005 ^c,d^
Return-to-Home ^a,b^	60 (23.2%)	104 (41.4%)	<0.001 ^c,d^

SLA, straight lateral approach; DAA, direct anterior approach. ^a^ Data expressed as frequency *n* (%). ^b^ Patients deceased during admission and not living independently at home prior to admission are excluded for analysis. ^c^ Fisher’s exact test. ^d^ Statistically significant difference.

**Table 3:** Perioperative outcomes and complication rates.

	SLA (*n* = 411)	DAA (*n* = 333)	*p*-Value
Duration of surgery (minutes) ^a^	68.6 ± 16.9	69.5 ± 14.5	0.445 ^d^
LOS after surgery (days) ^a,b^	5.80 ± 3.46	5.58 ± 4.09	0.440 ^d^
Duration of admission (days) ^a,b^	7.65 ± 3.86	7.53 ± 4.35	0.688 ^d^
Blood loss (mL) ^a^	286.5 ± 131.0 (*n* = 351)	295.4 ± 138.1 (*n* = 310)	0.398 ^d^
Haemoglobin drop (mmol/L) ^a^	−0.93 ± 0.71 (*n* = 406)	−0.93 ± 0.70 (*n* = 327)	0.967 ^d^
Complications ^c^			
• Conversion to THA	1 (0.2%)	2 (0.6%)	0.590 ^e^
• Dislocation	7 (1.7%)	3 (0.9%)	0.525 ^e^
• Periprosthetic fracture	2 (0.5%)	2 (0.6%)	1.00 ^e^
• PJI	6 (1.5%)	15 (4.5%)	0.024 ^e,f^
• Any type of revision	9 (2.2%)	16 (4.8%)	0.064 ^e^

SLA, straight lateral approach; DAA, direct anterior approach; LOS, length of stay; THA, total hip arthroplasty; PJI, periprosthetic joint infection. ^a^ Data are given as mean and standard deviation. ^b^ Patients deceased during admission are excluded for analysis. ^c^ Data expressed as frequency *n* (%). ^d^ Independent *t*-test. ^e^ Fisher’s exact test. ^f^ Statistically significant difference.

**Table 4:** Demographic of the subgroup analysis.

Characteristics	SLA FMS	DAA FMS	*p*-Value	SLA Katz ADL	DAA Katz ADL	*p*-Value
Number of HHAs	166	241	-	66	214	-
Age ^a^ (years)	81.2 ± 7.15	81.4 ± 7.40	0.833 ^c^	80.6 ± 7.62	81.3 ± 7.44	0.530 ^c^
Gender, Female ^b^	110 (66.3%)	159 (66.0%)	1.00 ^d^	46 (69.7%)	141 (65.9%)	0.654 ^d^
BMI ^a^ (kg/m^2^)	25.0 ± 3.49	25.7 ± 4.28	0.085 ^c^	25.8 ± 4.04	25.0 ± 3.55	0.100 ^c^
ASA grade ^b^			0.320 ^e^			0.223 ^e^
• ASA I	3 (1.9%)	4 (1.7%)		0 (0.0%)	4 (1.9%)	
• ASA II	74 (45.7%)	102 (42.3%)		28 (42.4%)	91 (42.5%)	
• ASA III	78 (48.1%)	131 (54.4%)		34 (51.5%)	115 (53.7%)	
• ASA IV	7 (4.3%)	4 (1.7%)		4 (6.1%)	4 (1.9%)	
Co-morbidities						
• COPD ^b^	18 (10.9%)	23 (9.5%)	0.738 ^d^	6 (9.1%)	21 (9.8%)	1.00 ^d^
• Dementia ^b^	17 (10.2%)	26 (10.8%)	1.00 ^d^	7 (10.6%)	22 (10.3%)	1.00 ^d^
• DM I/II ^b^	40 (24.1%)	42 (17.4%)	0.104 ^d^	12 (18.2%)	34 (15.9%)	0.705 ^d^
• Osteoporosis ^b^	37 (22.3%)	70 (29.0%)	0.138 ^d^	13 (19.7%)	60 (28.0%)	0.202 ^d^
Living at home prior to admission	136 (81.9%)	202 (83.8%)	0.687 ^d^	58 (87.9%)	182 (85.0%)	0.689 ^d^

SLA, straight lateral approach; DAA, direct anterior approach; FMS, Fracture Mobility Score; ADL, Activities of daily living; HHAs, hip hemiarthroplasties; BMI, body mass index; ASA, American Society of Anesthesiologist; COPD, Chronic obstructive pulmonary disease; DM I/II, diabetes mellitus—type I and II. ^a^ Data are given as mean and standard deviation. ^b^ Data expressed as frequency *n* (%). ^c^ Independent *t*-test. ^d^ Fisher’s exact test. ^e^ Chi-square test.

**Table 5:** Subgroup analysis of functional mobility and ADL-independence.

	Approach	Pre-Fracture Score	*p*-Value	Postoperative Score	*p*-Value	∆-Score	*p*-Value
FMS Mobility ^a^	SLA	1.98 ± 1.18	0.243 ^b^	2.57 ± 1.03	0.106 ^b^	0.60 ± 1.06	0.822 ^b^
	DAA	1.85 ± 1.07		2.40 ± 1.11		0.54 ± 1.10	
Katz ADL ^a^	SLA	5.26 ± 1.63	0.250 ^b^	4.53 ± 1.84	0.063 ^b^	−0.73 ± 1.57	0.036 ^b,c^
	DAA	5.05 ± 1.65		4.94 ± 1.66		−0.11 ± 1.60	

SLA, straight lateral approach; DAA, direct anterior approach; FMS, Fracture Mobility Score; ADL, Activities of daily living; The ∆-score is the difference between postoperative and pre-fracture scores (t1-0). ^a^ Data are given as mean and standard deviation. ^b^ Mann–Whitney U test. ^c^ Statistically significant difference.

## Data Availability

The data supporting the findings of this study are not publicly available due to restrictions related to patient privacy. Limited access to the data may be granted upon reasonable request to the corresponding author.

## Abbreviations

The following abbreviations are used in this manuscript:

HHA	Hip Hemiarthroplasty
FNF	Femoral Neck Fracture
SLA	Straight Lateral Approach
DAA	Direct Anterior Approach
PLA	Posterolateral Approach
FMS	Fracture Mobility Score
ADL	Activities of Daily Living
PJI	Periprosthetic Joint Infections
THA	Total Hip Arthroplasty
TFL	Tensor Fascia Lata
BMI	Body Mass Index
ASA	American Society of Anesthesiologist
COPD	Chronic Obstructive Pulmonary Disease
DM	Diabetes Mellitus
RtH	Return to Home
DoS	Duration of Surgery
PROM	Patient Reported Outcome Measures
DAIR	Debridement, Antibiotics, and Implant Retention

## References

[1] Florschutz A.V., Langford J.R., Haidukewych G.J., Koval K.J. (2015). Femoral neck fractures: Current management. J. Orthop. Trauma.

[2] Guzon-Illescas O., Perez Fernandez E., Crespí Villarias N., Quiroga Barrera A., Cuevas Cuerda E., Perez Rivas F.J., Nuñez Reiz A., González Ramírez A., Perez Fernandez M., Gil P. (2019). Mortality after osteoporotic hip fracture: Incidence, trends, and associated factors. J. Orthop. Surg. Res..

[3] Khalil K., Jamaleddine Y., Haj Hussein A., El-Jalbout R., Jad Z., El-Achkar M., El-Bitar C., Kassis N. (2025). Early postoperative mortality risk factors and five- and ten-year mortality rates after hip arthroplasty for femoral neck fracture. J. Clin. Med..

[4] Kim S.J., Park H.S., Lee D.W. (2020). Outcome of nonoperative treatment for hip fractures in elderly patients: A systematic review of recent literature. J. Orthop. Surg..

[5] Loggers S.A.I., Van Lieshout E.M.M., Joosse P., Verhofstad M.H.J., Willems H.C. (2020). Prognosis of nonoperative treatment in elderly patients with a hip fracture: A systematic review and meta-analysis. Injury.

[6] Jiang S., Yu T., Di D., Wang Y., Li W. (2024). Worldwide burden and trends of diabetes among people aged 70 years and older, 1990–2019: A systematic analysis for the Global Burden of Disease Study 2019. Diabetes Metab. Res. Rev..

[7] Miyamoto R.G., Kaplan K.M., Levine B.R., Egol K.A., Zuckerman J.D. (2008). Surgical management of hip fractures: An evidence-based review of the literature. I: Femoral neck fractures. J. Am. Acad. Orthop. Surg..

[8] Tol M.C.J.M., van Beers L.W.A.H., Willigenburg N.W., Gosens T., Van Embden D., Rhemrev S.J., Meylaerts S.A.G., Nunn T., Hogervorst M. (2021). Posterolateral or direct lateral approach for hemiarthroplasty after femoral neck fractures: A systematic review. Hip Int..

[9] Nogler M., Randelli F., Macheras G.A., Thaler M. (2021). Hemiarthroplasty of the hip using the direct anterior approach. Oper. Orthop. Traumatol..

[10] Unger A.C., Schulz A.P., Paech A., Jürgens C., Renken F.G. (2013). Modified direct anterior approach in minimally invasive hip hemi-arthroplasty in a geriatric population: A feasibility study and description of the technique. Arch. Orthop. Trauma Surg..

[11] Landelijke Registratie Orthopedische Interventies (LROI) (2025). Hip Hemiarthroplasty, Procedure Characteristics. https://www.lroi.nl/jaarrapportage/hip/hip-hemiarthroplasty/surgical-techniques/.

[12] Krassnig R., Prager W., Wildburger R., Hohenberger G.M. (2023). Direct anterior versus antero-lateral approach in hip joint hemiarthroplasty. Arch. Orthop. Trauma Surg..

[13] Kunkel S.T., Sabatino M.J., Kang R., Jevsevar D.S., Moschetti W.E. (2018). A systematic review and meta-analysis of the direct anterior approach for hemiarthroplasty for femoral neck fracture. Eur. J. Orthop. Surg. Traumatol..

[14] Khan I.A., Magnuson J.A., Arshi A., Krueger C.A., Freedman K.B., Fillingham Y.A. (2022). Direct anterior approach in hip hemiarthroplasty for femoral neck fractures: Do short-term outcomes differ with approach? A systematic review and meta-analysis. JBJS Rev..

[15] Manzo M.A., Hali K., Koucheki R., Wolfstadt J.I., Edwards T.C., Lex J.R. (2023). Complications and early recovery following hip hemiarthroplasty through the direct anterior approach: A systematic review and meta-analysis. Eur. J. Orthop. Surg. Traumatol..

[16] Carlson V.R., Ong A.C., Orozco F.R., Lutz R.W., Duque A.F., Post Z.D. (2017). The direct anterior approach does not increase return to function following hemiarthroplasty for femoral neck fracture. Orthopedics.

[17] Ladurner A., Schöfl T., Calek A.K., Zdravkovic V., Giesinger K. (2022). Direct anterior approach improves in-hospital mobility following hemiarthroplasty for femoral neck fracture treatment. Arch. Orthop. Trauma Surg..

[18] Bűcs G., Dandé Á., Patczai B., Gál T., Schmidt B., Horváth A., Than P. (2021). Bipolar hemiarthroplasty for the treatment of femoral neck fractures with minimally invasive anterior approach in elderly. Injury.

[19] Renken F., Renken S., Paech A., Wenzl M., Unger A., Schulz A.P. (2012). Early functional results after hemiarthroplasty for femoral neck fracture: A randomized comparison between a minimal invasive and a conventional approach. BMC Musculoskelet. Disord..

[20] Morrell A.T., Lindsay S.E., Schabel K., Parker M.J., Griffin X.L. (2025). Surgical approaches for inserting hemiarthroplasty of the hip in people with hip fractures. Cochrane Database Syst. Rev..

[21] Orth M., Osche D., Mörsdorf P., Grimm A., Scheuer C., Pohlemann T., Gercek E. (2023). Minimal-invasive anterior approach to the hip provides a better surgery-related and early postoperative functional outcome than conventional lateral approach after hip hemiarthroplasty following femoral neck fractures. Arch. Orthop. Trauma Surg..

[22] Ben Elyahu R., Ohana N., Agabaria E., Michaeli D., Shasha N., Rath E. (2023). Direct anterior vs. direct lateral approach total hip arthroplasty for displaced femoral neck fracture. J. Clin. Med..

[23] Visser L.C.E., Ponds N.H.M., Landman E.B.M., Bolink S.A.A.N. (2025). Transition from straight lateral to direct anterior approach in total hip arthroplasty: A retrospective single-centre study. Hip Int..

[24] Faldini C., Rossomando V., Brunello M., Mazzotti A., Panciera A., Viroli G., Evangelisti G., Ruffilli A., Traversari M., Vita F. (2024). Anterior minimally invasive approach (AMIS) for total hip arthroplasty: Analysis of the first 1000 consecutive patients operated at a high volume center. J. Clin. Med..

[25] Zampogna B., Papalia G.F., Parisi F.R., Vorini F., Torre G., Papalia R., Denaro V. (2023). Early return to activity of daily living after total hip arthroplasty: A systematic review and meta-analysis. Hip Int..

[26] Auffarth A., Resch H., Lederer S., Krismer M., Blauth M., Kapferer W. (2011). Does the choice of approach for hip hemiarthroplasty in geriatric patients significantly influence early postoperative outcomes? A randomized-controlled trial comparing the modified Smith-Petersen and Hardinge approaches. J. Trauma.

[27] McNally M., Sousa R., Wouthuyzen-Bakker M., Chen A.F., Soriano A., Vogely H.C., Clauss M., Higuera C.A., Trebše R. (2021). The EBJIS definition of periprosthetic joint infection. Bone Jt. J..

[28] Voeten S.C., Nijmeijer W.S., Vermeer M., Schipper I.B., Hegeman J.H., DHFA Taskforce study group (2020). Validation of the Fracture Mobility Score against the Parker Mobility Score in hip fracture patients. Injury.

[29] Roedl K.J., Wilson L.S., Fine J. (2016). A systematic review and comparison of functional assessments of community-dwelling elderly patients. J. Am. Assoc. Nurse Pract..

[30] McCabe D. (2019). Katz Index of Independence in Activities of Daily Living (ADL).

[31] Tsuda Y., Yasunaga H., Horiguchi H., Ogawa S., Kawano H., Tanaka S. (2015). Association between dementia and postoperative complications after hip fracture surgery in the elderly: Analysis of 87,654 patients using a national administrative database. Arch. Orthop. Trauma Surg..

[32] Bui M., Nijmeijer W.S., Hegeman J.H., Witteveen A., Groothuis-Oudshoorn C.G.M. (2024). Systematic review and meta-analysis of preoperative predictors for early mortality following hip fracture surgery. Osteoporos. Int..

[33] Wantonoro W., Kuo W.Y., Shyu Y.I.L. (2020). Changes in health-related quality of life for older persons with cognitive impairment after hip fracture surgery: A systematic review. J. Nurs. Res..

[34] Baxter S.N., Kelmer G.C., Brennan J.C., Johnson A.H., Turcotte J.J., King P.J. (2023). Acetabular total hip arthroplasty revision: A summary of operative factors, outcomes, and comparison of approaches. J. Arthroplast..

[35] Dockery D.M., Allu S., Glasser J., Antoci V., Born C.T., Garcia D.R. (2023). Comparison of periprosthetic joint infection rates in the direct anterior approach and non-anterior approaches to primary total hip arthroplasty: A systematic review and meta-analysis. Hip Int..

[36] Stone A.H., Sibia U.S., Atkinson R., Turner T.R., King P.J. (2018). Evaluation of the learning curve when transitioning from posterolateral to direct anterior hip arthroplasty: A consecutive series of 1000 cases. J. Arthroplast..

[37] Hu F., Jiang C., Shen J., Tang P., Wang Y. (2012). Preoperative predictors for mortality following hip fracture surgery: A systematic review and meta-analysis. Injury.

[38] Moldovan F., Moldovan L. (2025). The impact of total hip arthroplasty on the incidence of hip fractures in Romania. J. Clin. Med..

